# RETRACTION: Incidence and Management of Skin Lesions and Minor Wounds in Chronic Obstructive Pulmonary Disease Patients Undergoing Advanced Bronchodilator Therapy

**DOI:** 10.1111/iwj.70530

**Published:** 2025-04-18

## Abstract

**RETRACTION:** ShiC. and ZhangM. , “Incidence and Management of Skin Lesions and Minor Wounds in Chronic Obstructive Pulmonary Disease Patients Undergoing Advanced Bronchodilator Therapy,” International Wound Journal 21, no. 2 (2024): e14668, 10.1111/iwj.14668.PMC1083410042053029

The above article, published online on 01 February 2024, in Wiley Online Library (http://onlinelibrary.wiley.com/), has been retracted by agreement between the journal Editor in Chief, Professor Keith Harding; and John Wiley & Sons Ltd. Following an investigation by the publisher, all parties have concluded that this article was accepted solely on the basis of a compromised peer review process. The editors have therefore decided to retract the article. The authors did not respond to our notice regarding the retraction.

**RETRACTION:** C. Shi and M. Zhang , “Incidence and Management of Skin Lesions and Minor Wounds in Chronic Obstructive Pulmonary Disease Patients Undergoing Advanced Bronchodilator Therapy,” International Wound Journal 21, no. 2 (2024): e14668, 10.1111/iwj.14668.PMC1083410042053029

The above article, published online on 01 February 2024, in Wiley Online Library (http://onlinelibrary.wiley.com/), has been retracted by agreement between the journal Editor in Chief, Professor Keith Harding; and John Wiley & Sons Ltd. Following an investigation by the publisher, all parties have concluded that this article was accepted solely on the basis of a compromised peer review process. The editors have therefore decided to retract the article. The authors did not respond to our notice regarding the retraction.

